# Three-year trajectories of alcohol use among at-risk and among low-risk drinkers in a general population sample of adults: A latent class growth analysis of a brief intervention trial

**DOI:** 10.3389/fpubh.2022.1027837

**Published:** 2022-11-17

**Authors:** Sophie Baumann, Andreas Staudt, Jennis Freyer-Adam, Maria Zeiser, Gallus Bischof, Christian Meyer, Ulrich John

**Affiliations:** ^1^Department of Methods in Community Medicine, Institute for Community Medicine, University Medicine Greifswald, Greifswald, Germany; ^2^Faculty of Medicine, Institute and Policlinic of Occupational and Social Medicine, Technische Universität Dresden, Dresden, Germany; ^3^Institute for Medical Psychology, University Medicine Greifswald, Greifswald, Germany; ^4^German Centre for Cardiovascular Research, Partner Site Greifswald, Greifswald, Germany; ^5^Department of Psychiatry and Psychotherapy, University Lübeck, Lübeck, Germany; ^6^Department of Prevention Research and Social Medicine, Institute for Community Medicine, University Medicine Greifswald, Greifswald, Germany

**Keywords:** alcohol, trajectory, latent class, prevention, brief intervention, individualized feedback, general population, adults

## Abstract

**Background:**

Few studies have assessed trajectories of alcohol use in the general population, and even fewer studies have assessed the impact of brief intervention on the trajectories. Especially for low-risk drinkers, it is unclear what trajectories occur, whether they benefit from intervention, and if so, when and how long. The aims were first, to identify alcohol use trajectories among at-risk and among low-risk drinkers, second, to explore potential effects of brief alcohol intervention and, third, to identify predictors of trajectories.

**Methods:**

Adults aged 18-64 years were screened for alcohol use at a municipal registration office. Those with alcohol use in the past 12 months (*N* = 1646; participation rate: 67%) were randomized to assessment plus computer-generated individualized feedback letters or assessment only. Outcome was drinks/week assessed at months 3, 6, 12, and 36. Alcohol risk group (at-risk/low-risk) was determined using the Alcohol Use Disorders Identification Test–Consumption. Latent class growth models were estimated to identify alcohol use trajectories among each alcohol risk group. Sex, age, school education, employment status, self-reported health, and smoking status were tested as predictors.

**Results:**

For at-risk drinkers, a *light-stable* class (46%), a *medium-stable* class (46%), and a *high-decreasing* class (8%) emerged. The *light-stable* class tended to benefit from intervention after 3 years (Incidence Rate Ratio, IRR=1.96; 95% Confidence Interval, CI: 1.14–3.37). Male sex, higher age, more years of school, and current smoking decreased the probability of belonging to the *light-stable* class (*p*-values<0.05). For low-risk drinkers, a *very light-slightly increasing* class (72%) and a *light-increasin*g class (28%) emerged. The *very light-slightly increasing* class tended to benefit from intervention after 6 months (IRR=1.60; 95% CI: 1.12–2.28). Male sex and more years of school increased the probability of belonging to the *light-increasing* class (*p*-value < 0.05).

**Conclusion:**

Most at-risk drinkers did not change, whereas the majority of low-risk drinkers increased alcohol use. There may be effects of alcohol feedback, with greater long-term benefits among persons with low drinking amounts. Our findings may help to identify refinements in the development of individualized interventions to reduce alcohol use.

## Introduction

Alcohol use causes increased risk of morbidity and death (1, 2). This effect has usually been attributed to high drinking amounts commonly referred to as “at-risk” alcohol use. The National Institute in Alcohol Abuse and Alcoholism (NIAAA) defines at-risk alcohol use as consuming more than 14 drinks of 14 g alcohol each per week or more than 4 drinks on any day for males and more than seven drinks per week or three drinks on any day for females, respectively (3). Lower drinking amounts were considered acceptable for a long time, and “low-risk” drinkers have been excluded from interventions to reduce alcohol use. This contradicts the public health approach to decrease alcohol use in the general population in order to reduce alcohol-attributable health disorders (4). Previous studies have observed a higher risk of disease and mortality for alcohol abstainers compared to low-risk drinkers (5). Meanwhile, evidence is mounting that misclassification of former or occasional drinkers as abstainers and other confounders favoring low-risk drinkers over abstainers are responsible for the observed J-shape function relating alcohol use to disease and mortality (6, 7). Even low drinking amounts have been found to be associated with increased risk of adverse brain and cardiovascular outcomes as well as prevalent cancers, particularly neoplasms of the upper and lower digestive tract and the female breast (8–11). Thus, it is important to understand and address alcohol use in the population as a whole, including persons with low drinking amounts. As alcohol use is unlikely to be characterized as a static phenomenon (12), trajectories are important but have not been thoroughly investigated so far.

There are only few longitudinal studies describing the trajectories of alcohol use in the adult population as a whole. A cohort study among U.S. general population adults found that 81% of the participants identified as low-risk drinkers (< 20 grams of pure alcohol per day for females; < 40 for males) were still at low-risk after 3 years, and 13% had become abstinent (13). Data of the same study revealed that among the participants with at-risk alcohol use (> 14 drinks per week or > 4 drinks per occasion for males; > 7 and > 3, respectively, for females), 27% had become low-risk drinkers or abstinent (14).

Limitations of the research so far include that trajectories of alcohol use were mostly estimated under the assumption that all persons in the sample come from a single population. Emerging evidence from the literature indicates that there are distinct subpopulations each comprised of persons with similar trajectories over time (15). Different trajectories, in turn, may have different etiological pathways and consequences, and persons with different trajectories may therefore require different approaches to help them change (16). Indeed, the sparse available literature points toward the existence of different patterns of response to brief alcohol interventions, with better outcomes for persons who drink low amounts of alcohol (15, 17). The currently available evidence on this issue is limited to persons identified as at-risk drinkers. Little is known about low-risk drinkers. A better understanding about different trajectories of alcohol use and mechanisms which determine the trajectories can be used to inform the development of individualized interventions tailored to the special needs of at-risk and low-risk drinkers.

Latent class growth models (LCGMs) are a popular method to uncover different growth trajectories in longitudinal observational studies. This paper provides an application of such modeling approach to a randomized trial of a brief alcohol intervention. This trial was designed to test the efficacy of computer-generated individualized feedback letters and is one of the few that addressed all adults from a general population sample irrespective of quantity, frequency, and consequences of their alcohol use, and followed them long-term (over 3 years). This allows us to examine long-term trajectories of alcohol use with and in absence of brief intervention as well as among at-risk drinkers and among low-risk drinkers. The latter in particular represent a so far understudied but important group of persons in the field of brief intervention research.

This study aimed first, to uncover different trajectories of alcohol use over 3 years among at-risk and among low-risk drinkers in a general population sample of adults. The second aim was to explore potential effects of a brief alcohol intervention on the different trajectories of alcohol use. The third aim was to examine factors that predict the different trajectories.

## Materials and methods

The current study presents 3-year data from the randomized controlled trial entitled “Testing a PRoactive expert system INTervention to prevent and to quit at-risk alcohol use” (PRINT). The primary aim of the PRINT trial was to test the effect of computer-generated individualized feedback letters on the number of drinks per week in a general population sample of adults with alcohol use. Participants were individually randomized to one of two parallel groups. The intervention group and the control group received assessment plus computer-generated individualized feedback letters or assessment only at baseline, month 3, and month 6, respectively. Both groups were followed at year 1. The PRINT trial was prospectively registered at the German Clinical Trials Register (DRKS00014274, date of registration: 12 March 2018) and approved by the ethics committee of University Medicine Greifswald (BB 147/15). The protocol was published on 9 July 2018 (18). We received renewal funding for additional follow-ups to investigate the maintenance of potential intervention effects in the longer term. These follow-ups were approved by the ethics committees of University Medicine Greifswald (BB 053/19) and TU Dresden (SR-EK-272062020). Primary and secondary outcome data has been published elsewhere (19, 20). The current analysis is of exploratory nature, and we report the findings as ancillary data.

### Participants and procedure

From April to June 2018, participants were recruited proactively at the registry office in Greifswald, Mecklenburg-West Pomerania, Germany. Every adult resident in Germany needs to contact this public authority at regular intervals, e.g., for registration, identification card and passport issues, or vehicle admission. During the entire opening hours, study assistants invited all clients aged 18–64 years appearing in the waiting area to take part in a self-administered computer-based survey containing the eligibility screening for the subsequent trial. Clients already approached, with notable cognitive impairment or a physical condition that prevent trial participation, with insufficient German language or reading skills, or employed at the conducting research institute were excluded. Those who answered yes to the question “Did you consume any alcohol in the previous 12 months?” were eligible and invited to take part in the trial. Those having no telephone or permanent address were excluded. All trial participants provided written informed consent and received a voucher of 5 euros.

Participants were assigned to intervention or assessment only using a computer-generated list of random numbers. Simple randomization with a 1:1 allocation ratio was used. Study assistants responsible for participant recruitment were not informed about group allocation. Group allocation was not revealed to participants until they received feedback or not.

One- and 3-year follow-ups were conducted *via* structured computer-assisted telephone interviews from April to July 2019 and from April to July 2021, respectively. Outcome assessors were not informed about group allocation. Ten telephone contact attempts were made before participants received an equivalent questionnaire by email or postal mail, with up to two reminders. Participants received a voucher of 5 euros for each follow-up assessment.

### Sample size calculation

The variable drinks per week was expected to follow a negative binomial distribution. For μ = 10 drinks in the control group and μ = 8.5 in the intervention group, a dispersion parameter of 1.0, 80% power, and 5% significance level, 659 participants per group are required. Considering a 20% dropout, our calculations yielded a total sample size of *N* = 1,648.

### Study groups

#### Intervention

The intervention was based on expert system technology that automatically generated individualized feedback based on participant data (21). It included three intervention contacts (at baseline, month 3, and month 6). For each intervention contact, participants were proactively approached by study assistants for assessment of demographics, alcohol use, and motivational constructs according to the transtheoretical model of behavior change (TTM) (22, 23). Data were assessed either through self-administered questionnaires provided on tablet computers (at baseline) or through structured computer-assisted telephone interviews (at months 3 and 6). The expert system software analyzed data in comparison to general population data, selected feedback modules, and generated a feedback letter that was sent to the participant by postal mail.

The content and number of feedback elements were tailored according to alcohol risk group (for method of calculation, see Measures subsection). Low-risk drinkers received a 2-page letter including reinforcement of drinking within low-risk limits, the information that alcohol use can produce problems even within these limits, and feedback regarding their alcohol use in comparison to data about alcohol use in the general population of the same sex and age. At-risk drinkers received a 3- to 4-page letter including feedback regarding their alcohol use and TTM constructs in comparison to data from other general population samples about alcohol use and TTM constructs of the same sex and age. Persons with possible alcohol use disorder (AUD) additionally received feedback on their perceived AUD symptoms and information on local alcohol treatment services. For a more detailed description of the intervention, please see elsewhere (19).

#### Control

Participants in the control group received no feedback. They underwent the same assessment procedure as those in the intervention group except that no motivational constructs according to the TTM were assessed as the questions may elicit thinking about behavior change.

### Measures

#### Alcohol risk group

Alcohol risk group was calculated based upon baseline scores of the Alcohol Use Disorders Identification Test (AUDIT) and its short form, the AUDIT-C (4, 24, 25): at-risk (AUDIT-C score 4–12 for females and 5–12 for males, but AUDIT scores ≤ 19), low-risk (AUDIT-C score 1–3 for females and 1–4 for males), and possible AUD (AUDIT score 20–40).

#### Alcohol use

The number of drinks per week was assessed at baseline, months 3 and 6, and years 1 and 3. It was calculated based on self-reports of frequency and quantity of alcohol use in the past 30 days: “How often did you have an alcoholic drink: never (frequency multiplier: 0 drinking days per month), once (1), 2–4 times (3), 2–3 times per week (10), or ≥ 4 times per week (22)?” and “How many drinks did you typically have on a drinking day?”. A drink was defined as 10 grams of pure alcohol equivalent to 0.25–0.3 l beer, 0.1–0.15 l wine or sparkling wine, or 4 cl spirits. To determine the average number of drinks per week, the frequency was multiplied by the number of drinks, and the total was divided by the number of weeks in a month and rounded down to the nearest integer.

#### Predictor variables

Socio-demographic variables were assessed at baseline and included sex (female, male), age, years of school education with German school types classified as < 10 years, 10–11 years, or ≥ 12 years, and employment status categorized as full-time employed, part-time employed or unemployed, education, or other (i.e., retiree, on maternity or parental leave, homemaker). Self-reported health was assessed using a single item: “Would you say your health in general is: excellent, very good, good, fair, or poor?” Due to small cell occupation, “fair” and “poor” were merged into one category. Smoking status was assessed by the question: “Do you smoke currently?” and four possible answers: “No, I have never smoked” (never smoker), “No, I am not a smoker now” (former smoker), “Yes, I smoke daily” (current daily smoker), and “Yes, I smoke sometimes” (current less than daily smoker).

### Statistical analyses

**Figure 1:**
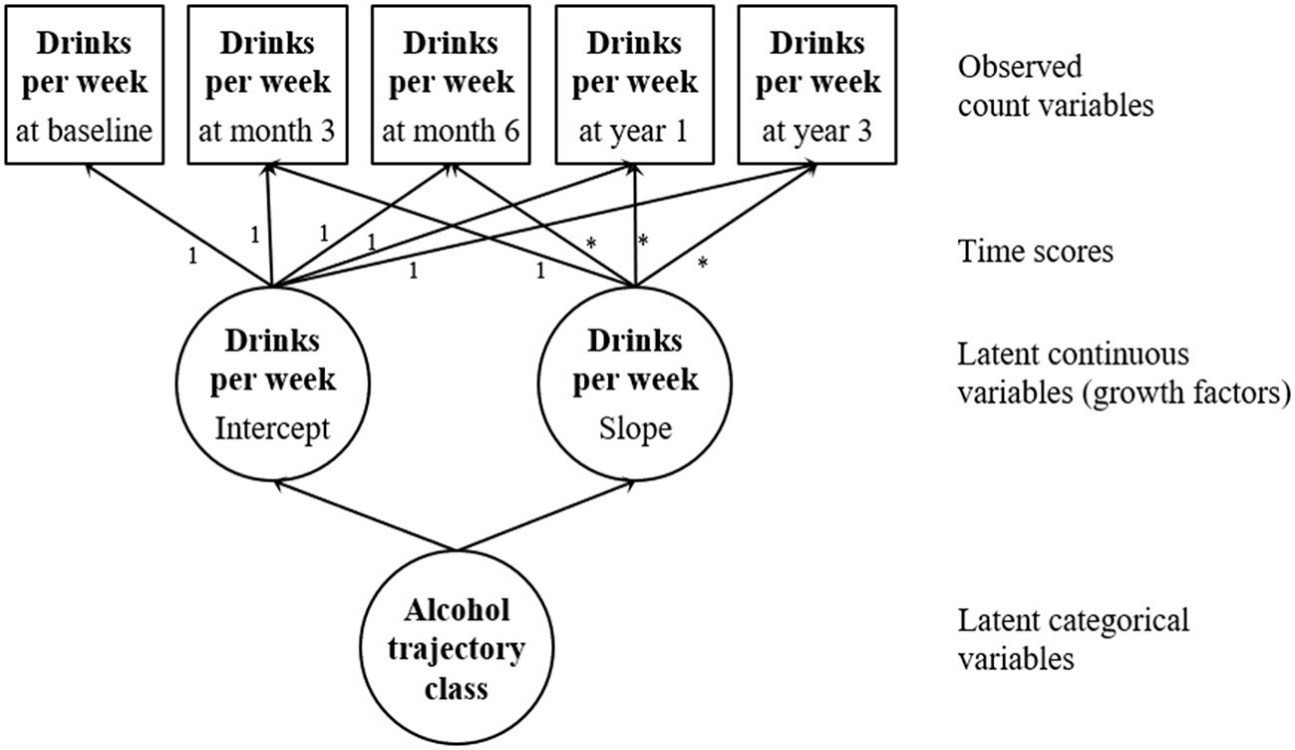
Latent class growth model.

LCGMs were estimated using Mplus version 8.8 (26) to identify latent trajectory classes for each alcohol risk group. Separate models for at-risk and low-risk drinkers allowed us first, to take account of that dose and content of the intervention differ between alcohol risk groups [please see subsection 2.3, or (19) for more details], and second, to generate new knowledge concerning a group of persons (low-risk drinkers) that has mostly been excluded from brief intervention studies. A maximum likelihood estimator with robust standard errors using a numerical integration algorithm was applied. It produces accurate model parameter under a missing at random assumption and maximizes power using all available data (intention-to-treat principle). As shown in Figure 1, trajectories of alcohol use were captured by two latent growth factors representing the initial level of alcohol use (intercept) and the rate of change over time (slope). The growth factors were based on five observed indicators representing the number of drinks per week at baseline, month 3, month 6, year 1, and year 3. Indicators were regressed on the growth factors using a negative binomial model. Time scores were treated as parameters that need to be estimated to capture non-linear trajectories.

Data were analyzed in three steps. First, the development of alcohol use in the control group was studied to establish “normative” trajectories in absence of intervention. It was assumed that the intervention may produce changes in trajectories within trajectory classes without affecting class membership itself (16). That is, intervention effects may differ across classes as indicated by study group differences in the rate of change in alcohol use, but the model did not allow transitions between classes as a result of the intervention. Therefore, the number of classes was determined using the control group, and it was assumed that the number of classes identified also hold in the intervention group. The determination of the optimal number of classes was guided by information criteria, theoretical interpretability, and class size. Akaike information criterion (AIC), Bayesian information criterion (BIC), and sample-size adjusted BIC (27–29) were reported, with lower values indicating better fit. The Lo-Mendell-Rubin adjusted likelihood ratio test (LMR-LRT) was estimated to compare the estimated model with a model with one class less (26, 30). *P*-values < 0.5 indicate that the model with one less class fits the data not as well as the estimated model. Classification diagnostics were examined but not used for model selection. Entropy values and average conditional class probabilities of correct class-classification were obtained (31, 32). Values close to 1 indicate clear classification (16).

Second, alcohol use trajectories in the control and the intervention group were jointly modeled to explore whether persons in different trajectory classes benefitted differently from intervention. Because of randomization, study groups were assumed to be equivalent on alcohol use at baseline. Class membership was not regressed on study group, so that effects would be attributable to the intervention and not transitions between classes (16). Due to the exploratory nature of this study, class-specific intervention effects were explored graphically and using relative effect sizes. Incidence rate ratios (IRRs) indicating study group differences with regard to change in drinks per week from baseline to follow-up (net change) with 95% confidence intervals (CIs) were calculated. IRR values of 1.22 (0.82), 1.86 (0.54), and 3.0 (0.33) were used as benchmarks for quantifying small, medium, and large beneficial [adverse] effects of the intervention (33).

Third, the latent trajectory class variable was regressed on a set of baseline covariates (sex, age, school education, employment status, self-reported health, and smoking status) to investigate potential predictors of trajectory classes.

## Results

### Study sample

**Figure 2:**
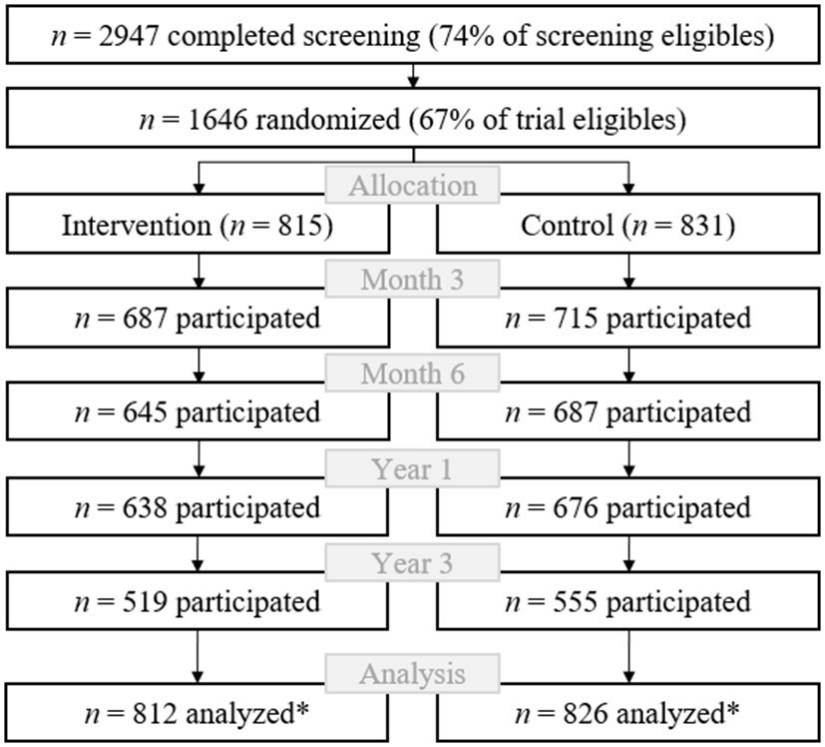
Participant flow. *n = 8 participants identified as having possible alcohol use disorder were excluded.

Of the registry office clients who met the inclusion criteria for the eligibility screening, 2947 (74%) completed the screening assessment (Figure 2). Of the eligibles, 1,646 (67%) participated in the PRINT trial. The 3- and 6-month assessments were completed by 1,406 (85%) and 1335 (81%) participants, respectively. The 1-year follow-up participation rate was 80% (*n* = 1314). Of the 1,581 (96%) participants who gave their consent for recontact in case of renewal funding, 1,074 (68%) participated in the 3-year follow-up. For a detailed flow chart, please see elsewhere (19). Among the baseline participants, 533 (34%) were identified as at-risk drinkers, 1,085 (66%) were identified as low-risk drinkers, and 8 (< 1%) were identified as having possible AUD. This group was excluded from analysis due to few observations.

The final sample analyzed in this study (*n* = 1638) was composed of 919 (56%) females and 719 (44%) males with a mean age of 31.0 years (SD = 10.8). Among the sample, 1067 (65%) had 12 or more years of school, 471 (29%) 10–11 years of school, and 100 (6%) less than 10 years of school. The mean alcohol use was 3.9 drinks per week (SD = 5.4) for at-risk drinkers and 0.7 drinks (SD = 1.7) for low-risk drinkers. For detailed baseline sample characteristics, please see elsewhere Bauman et al. (19) and Enders et al. (34).

### Classes of alcohol use trajectories among at-risk drinkers

AIC, BIC, and aBIC decreased with increasing number of classes (Table 1). The estimation of models specifying more than three classes led to very small class sizes and empirical under-identification. The LMR-LRT indicates that the two-class model is optimal. However, adding a third class resulted in the addition of a qualitatively distinct and meaningful class of persons exceeding the weekly drinking limit for males. Therefore, we selected the three-class model for further analysis. Entropy was 0.74 and average latent class posterior probabilities ranged between 0.87 and 0.96.

**Table 1:** Model fit and diagnostic criteria for alternative trajectory models for the control group separately for at-risk drinkers and low-risk drinkers.

	**Model fit criteria**					**Diagnostic criteria**		
**Models**	**LL (# free parameters)**	**AIC**	**BIC**	**aBIC**	**LMR-LRT**	**Smallest class, *n* (%)**	**Entropy**	**ALCPP**
At-risk drinkers (*n =* 286)								
1 class	−2969.61 (10)	5959.22	5995.78	5964.07	—	286 (100)	—	—
2 classes	−2792.08 (16)	5616.16	5674.66	5623.92	< 0.001	51 (18)	0.86	0.93–0.97
3 classes	−2749.99 (22)	5543.97	5624.41	5554.64	0.665	23 (8)	0.74	0.87–0.96
4 classes^*^	−2719.92 (28)	5495.85	5598.22	5509.43	0.038	3 (< 1)	0.77	0.84–0.93
5 classes	−2706.52 (34)	5481.03	5605.34	5497.52	0.106	3 (< 1)	0.77	0.82–0.98
Low-risk drinkers (*n =* 540)								
1 class	−3084.34 (10)	6188.68	6231.59	6199.85	—	540 (100)	—	—
2 classes	−2874.76 (16)	5781.52	5850.18	5799.39	< 0.001	151 (28)	0.72	0.91–0.93
3 classes	−2830.96 (22)	5705.92	5800.34	5730.50	0.136	88 (16)	0.65	0.81–0.89
4 classes	−2818.62 (28)	5693.24	5813.40	5724.52	0.259	4 (< 1)	0.71	0.81–0.95
5 classes^*^	−2805.25 (34)	5678.50	5824.41	5716.49	0.092	11 (< 1)	0.72	0.80–0.95

LL, log-likelihood; AIC, Akaike information criterion; BIC, Bayesian information criterion; aBIC, sample-size adjusted BIC; LMR-LRT, Lo-Mendell-Rubin adjusted likelihood ratio test (p-value); ALCPP, average latent class posterior probability.

* Singular information matrix required some model parameters to be fixed by the program and they were not estimated.

**Figure 3:**
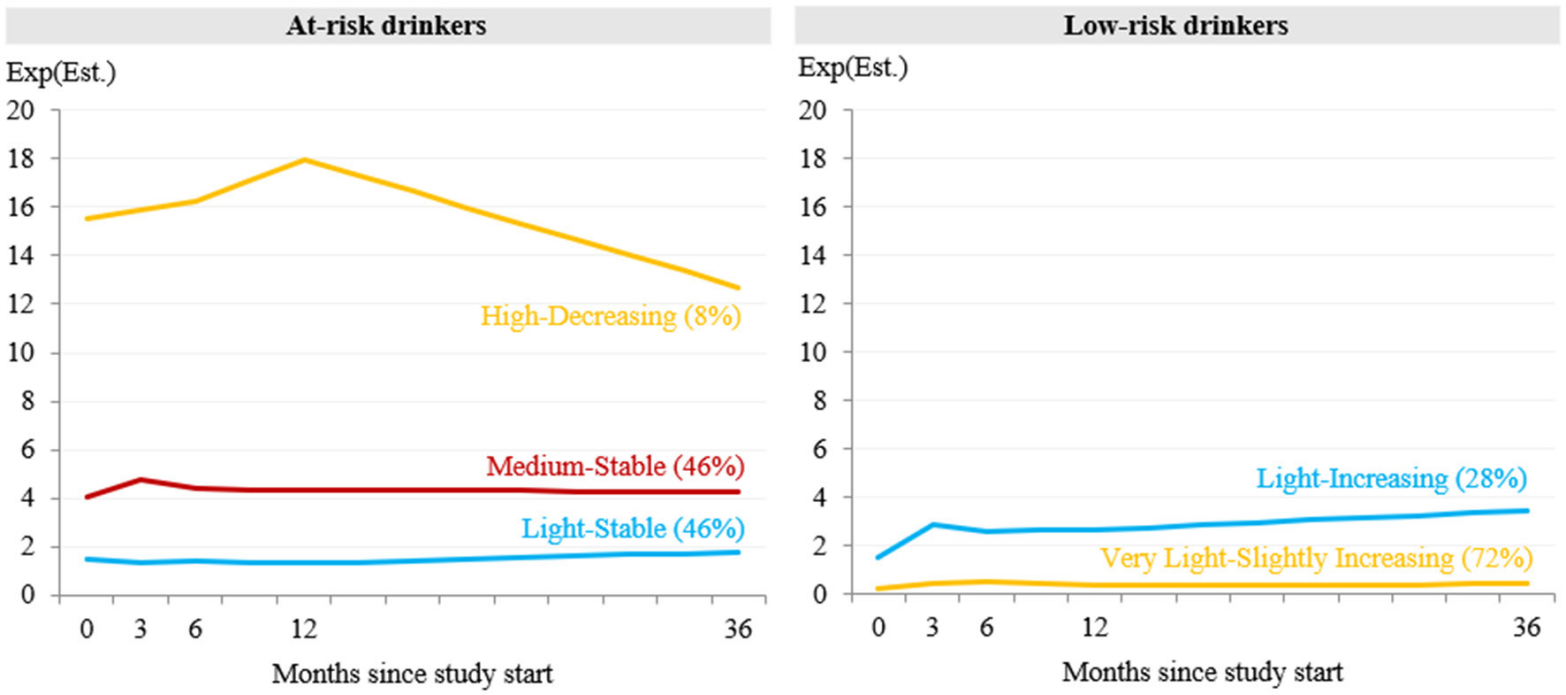
Estimated mean trajectories of drinks per week over 3 years in absence of intervention for the models with 3 classes for at-risk drinkers and 2 classes for low-risk drinkers. Est = Estimate.

In the absence of intervention, the two largest classes showed a stable alcohol use trajectory over time (Figure 3). Persons in the *medium-stable* class (*n* = 132, 46%) had on average 4 to 5 drinks per week. The *light-stable* class (*n* = 131, 46%) included persons whose alcohol use ranged on average between 1 to 2 drinks per week at any measurement point. The smallest class (*n* = 23, 8%) was labeled *high-decreasing* class and comprised persons who increased alcohol use on average from 16 drinks per week at baseline to 18 drinks per week at year 1 and then decreased it to 13 drinks per week at year 3.

**Figure 4:**
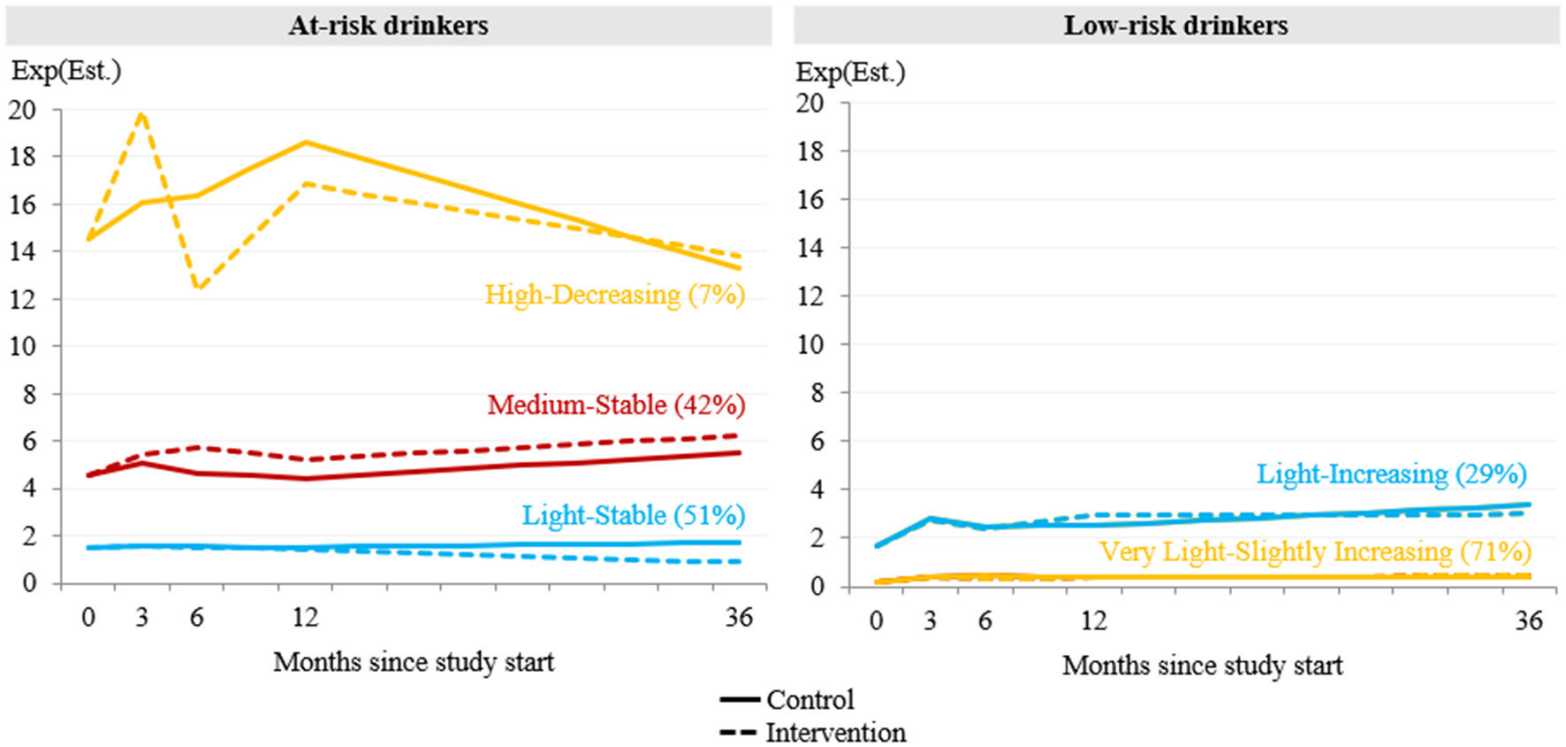
Jointly estimated mean trajectories of drinks per week over 3 years for the control and the intervention group (*N* = 1638). Est, Estimate.

In the joint analyses of the control and intervention group, the inclusion of covariates did not substantially alter the results. Therefore, results from the model with covariates are reported here (for results of the model without covariates, please see Supplementary Figure S1 and Table S1). It was found that persons in the *light-stable* class who received alcohol feedback reduced alcohol use over time, from on average 1.5 drinks at baseline to 0.9 drinks at year 3 (Figure 4). As shown in Table 2, the study group difference at year 3 was significant and represents a medium-sized beneficial effect of the intervention (IRR = 1.96, CI_95%_: 1.14–3.37). Persons in the *medium-stable* class who received feedback increased alcohol use on average from 4.5 to 6.2 drinks. Analysis revealed small but not statistically significant adverse effects at month 6 (IRR = 0.81, CI_95%_: 0.58–1.14) and at year 3 (IRR = 0.75, CI_95%_: 0.47–1.17). Persons in the *high-decreasing* class with feedback increased their alcohol use at month 3 and then tended to drink less than those without feedback. Analysis revealed a small but not statistically significant adverse effect at month 3 (IRR = 0.81, CI_95%_: 0.51–1.28) and a small but not statistically significant beneficial effect at month 6 (IRR = 1.33, CI_95%_: 0.68–2.61).

**Table 2:** Study group differences (net changes) in drinks per week over 3 years according to trajectory class.

	**At-risk drinkers**						**Low-risk drinkers**			
	**Light-stable**		**Medium-stable**		**High-decreasing**		**Very light-slightly increasing**		**Light-increasing**	
	**IRR**	**(95% CI)**	**IRR**	**(95% CI)**	**IRR**	**(95% CI)**	**IRR**	**(95% CI)**	**IRR**	**(95% CI)**
Month 3	0.97	(0.72–1.32)	0.93	(0.69–1.23)	0.81	(0.51–1.28)	0.94	(0.70–1.28)	1.02	(0.75–1.38)
Month 6	1.01	(0.71–1.44)	0.81	(0.58–1.14)	1.33	(0.68–2.61)	**1.60**	**(1.12–2.28)**	1.06	(0.80–1.40)
Year 1	1.08	(0.75–1.57)	0.85	(0.64–1.11)	1.11	(0.42–2.90)	1.04	(0.71–1.54)	0.87	(0.66–1.13)
Year 3	**1.96**	**(1.14–3.37)**	0.75	(0.47–1.17)	0.96	(0.84–1.89)	0.84	(0.58–1.20)	1.12	(0.79–1.60)

IRR, incidence rate ratio; CI, confidence interval. Trajectory classification quality for at-risk drinkers: entropy = 0.79, average latent class posterior probability range = 0.90–0.96. Trajectory classification quality for low-risk drinkers: entropy = 0.76, average latent class posterior probability range = 0.92–0.93. The bold values indicate study group differences with value of *p* < 0.05.

### Classes of alcohol use trajectories among low-risk drinkers

Three trajectory classes were suggested by the BIC. AIC and aBIC decreased with increasing number of trajectory classes. Again, models with more than three classes consisted of small classes with few observations. The LMR-LRT indicates that the model with two classes fits the data as well as the model with three classes. Therefore and because two of the estimated mean trajectories within the three-class model were very similar and their separation may not be meaningful (see Supplementary Figure S2), we selected the two-class model for further analysis. Entropy was 0.72 and average latent class posterior probabilities ranged between 0.91 and 0.93.

Persons in the largest trajectory class (*n* = 389, 72%) increased their average alcohol use per week from 0.2 drinks at baseline to 0.4 drinks at month 3 and then remained stable over time. This class was labeled *very light-slightly increasing*. The *light-increasing* class (*n* = 151, 28%) was composed of persons who increased alcohol use from 1.5 to 3.4 drinks per week over 3 years.

Although within-class trajectories were similar between study groups, alcohol feedback had a small and statistically significant effect on drinks per week at month 6 in the *very light-slightly increasing* class (IRR = 1.60, CI_95%_: 1.12–2.28).

### Predictors of trajectory class membership

Table 3 provides baseline sample characteristics by trajectory class. For the at-risk group, persons in the *medium-stable* class were more likely to be male, current daily or less than daily vs. never smoker, more often had ≥ 12 vs. < 10 years of school, and were more likely to be retired, on maternity or parental leave, or homemaker vs. full-time employed compared to the *light-stable* class (Table 4). Persons in the *high-decreasing* class were more likely to be male, older, and current daily or less than daily vs. never smoker compared to the *light-stable* class. For the low-risk group, persons in the *light-increasing* class were more likely to be male, more often had ≥ 12 vs. < 10 years of school, and (by trend) were more likely to be current daily vs. never smoker compared to the *very light-slightly increasing* class.

**Table 3:** Baseline sample characteristics by trajectory class (N = 1638).

	**At-risk drinkers**			**Low-risk drinkers**	
	**Light-stable**	**Medium-stable**	**High-decreasing**	**Light-increasing**	**Very light-slightly increasing**
Age in years	27.5 (9.3)	29.3 (10.2)	33.7 (13.8)	32.6 (10.9)	32.0 (11.0)
**Sex**					
Female	237 (84.0)	94 (40.5)	4 (10.3)	55 (17.5)	529 (68.6)
Male	45 (16.0)	138 (59.5)	35 (89.7)	259 (82.5)	242 (31.4)
**School education**					
< 10 years	17 (6.0)	6 (2.6)	5 (12.8)	11 (3.5)	61 (7.9)
10–11 years	74 (26.2)	38 (16.4)	8 (20.5)	93 (29.6)	248 (33.5)
≥ 12 years	191 (67.7)	188 (81.0)	26 (66.7)	210 (66.9)	452 (58.6)
**Employment status**					
Full-time employed	88 (31.2)	83 (35.8)	17 (43.6)	157 (50.0)	340 (44.1)
Unemployed/ part-time employed	80 (28.4)	72 (31.0)	16 (41.0)	70 (22.3)	181 (23.5)
Education	106 (37.6)	71 (30.6)	5 (12.8)	74 (23.6)	190 (24.6)
Other^*^	8 (2.8)	6 (2.6)	1 (2.6)	13 (4.1)	60 (7.8)
**Smoking status**					
Never smoker	145 (51.4)	86 (37.1)	6 (15.4)	164 (52.2)	464 (60.2)
Former smoker	49 (17.4)	29 (12.5)	6 (15.4)	57 (18.2)	107 (13.9)
Current less than daily smoker	28 (9.9)	48 (20.7)	7 (18.0)	28 (8.9)	56 (7.2)
Current daily smoker	60 (21.3)	69 (29.7)	20 (51.2)	65 (20.7)	144 (18.7)
**Self-reported health**					
Excellent	26 (9.2)	22 (9.5)	4 (10.3)	24 (7.6)	52 (6.7)
Very good	112 (39.7)	85 (36.6)	16 (41.0)	145 (46.2)	286 (37.1)
Good	125 (44.3)	112 (48.3)	15 (38.4)	123 (39.2)	377 (48.9)
Fair/ poor	19 (6.7)	13 (5.6)	4 (10.3)	22 (7.0)	56 (7.3)

Data are numbers (percent) or means (standard deviations) for age.

* Retired, on maternity or parental leave, homemaker.

**Table 4:** Results from multinomial logistic regressions to predict latent trajectory class membership for at-risk drinkers and for low-risk drinkers.

	**At-risk drinkers (*n =* 553)**						**Low-risk drinkers (*n =* 1085)**		
	**Medium-stable** ***vs. light-stable***			**High-decreasing** ***vs. light-stable***			**Light-increasing** ***vs. very light-slightly increasing***		
	**RRR**	**95% CI**	***p*** **-value**	**RRR**	**95% CI**	***p*** **-value**	**RRR**	**95% CI**	***p*** **-value**
Age in years	1.02	0.99–1.06	0.125	**1.08**	**1.02–1.13**	**0.005**	1.01	0.99–1.03	0.301
**Sex**									
*Female*	*1.00*			*1.00*			*1.00*		
Male	**8.17**	**4.28–15.57**	**<0.001**	**59.62**	**15.89–223.69**	**<0.001**	**9.33**	**6.17–14.13**	**<0.001**
**School education**									
*< 10 years*	*1.00*			*1.00*			*1.00*		
10–11 years	2.18	0.40–11.74	0.365	0.52	0.09–3.03	0.464	1.86	0.78–4.42	0.159
≥ 12 years	**10.39**	**1.86–57.99**	**0.008**	3.64	0.60–22.24	0.161	**3.03**	**1.24–7.45**	**0.015**
**Employment status**									
*Unemployed/ part-time employed*	*1.00*			*1.00*			*1.00*		
Full-time employed	1.19	0.56–2.24	0.751	0.74	0.25–2.21	0.592	0.69	0.42–1.13	0.142
Education	0.82	0.41–1.63	0.569	0.53	0.09–3.19	0.486	0.94	0.53–1.69	0.847
Other^*^	**6.19**	**1.58–24.23**	**0.009**	3.26	0.30–35.54	0.332	0.53	0.23–1.23	0.140
**Smoking status**									
*Never smoker*	*1.00*			*1.00*			*1.00*		
Former smoker	0.63	0.24–1.67	0.355	1.34	0.32–5.72	0.689	1.45	0.83–2.50	0.190
Current less than daily smoker	**2.94**	**1.38–6.25**	**0.005**	**5.33**	**1.17–24.23**	**0.030**	1.63	0.82–3.26	0.165
Current daily smoker	**2.27**	**1.06–4.86**	**0.036**	**8.81**	**1.90–40.76**	**0.005**	1.57	0.94–2.62	0.089
**Self-reported health**									
*Excellent*	*1.00*			*1.00*			*1.00*		
Very good	1.16	0.44–3.01	0.767	0.77	0.09–6.94	0.417	1.08	0.52–2.25	0.837
Good	1.31	0.50–3.45	0.582	0.47	0.08–2.93	0.667	1.03	0.49–2.17	0.949
Fair/ poor	1.03	0.18–6.02	0.974	0.53	0.03–9.72	0.689	1.31	0.50–3.45	0.588

RRR, relative risk ratio; CI, confidence interval.

* Retired, on maternity or parental leave, homemaker. The bold values indicate differences with value of *p* < 0.05.

## Discussion

This study provides new insights into the development of alcohol use over time in a general population sample of adult alcohol users and the potential impact of a brief alcohol intervention on the different developmental courses. Three main findings emerged. First, three trajectories of alcohol use among at-risk drinkers and two trajectories among low-risk drinkers were uncovered that were not represented by the average developmental course over time in the whole sample. Second, subgroups with different types of alcohol use trajectories tended to respond differently well to individualized alcohol feedback. Third, for both at-risk drinkers and low-risk drinkers, trajectories characterized by higher amounts of alcohol use were associated with male sex, high level of school education, and smoking.

For the at-risk group, control group data indicate three distinct subpopulations of persons with similar trajectories of alcohol use over 3 years. Nine in ten participants showed a quite stable drinking trajectory and were allocated either to the *light-stable* or to the *medium-stable* class. The proportion of at-risk drinkers who did not change was higher than observed in the National Epidemiologic Survey on Alcohol and Related Conditions (NESARC) study (13, 14), possibly due to cultural differences in social norms toward alcohol use among German vs. U.S. adults. Germany is a high per capita consumption country with a liberal alcohol policy and may be characterized by a permissive drinking culture (35–37). In the U.S., the attitude toward alcohol use may be more ambivalent due to conflicting co-existing value structures (36, 38). A small class of participants with high drinking amounts had decreased their alcohol use in absence of intervention (*high-decreasing*). Higher drinking amounts are likely to be associated with higher motivation to change alcohol use, for example due to more perceived negative consequences (39).

For the low-risk group, control group data indicate two distinct trajectories of alcohol use. The majority of participants engaged in a trajectory characterized by a very low level of alcohol use that was slightly increased over time (*very light-slightly increasing*). The second trajectory class was similar to the first, but with a slightly higher initial level and a steeper increase of alcohol use over time (*light-increasing*). Notably, the level of alcohol use at baseline was estimated to be almost identical for the *light-increasing* class (low-risk drinkers) and the *light-stable* class (at-risk drinkers), but it was increased in the former and remained stable in the latter class. The question arises whether the estimated curves within the classes actually represent distinct trajectories, or whether they only differ in that participants identified as at-risk drinkers had a heavy episodic drinking occasion at baseline whereas those identified as low-risk drinkers had not. Parts of the increase in alcohol use seen in baseline low-risk drinkers may be due to regression to the mean (40).

Our finding that the majority of low-risk drinkers increased their alcohol use after 3 years is contrary to what has been found in U.S. general population adults. In the NESARC cohort, only 5 to 15% of the low-risk drinkers had increased their alcohol risk level (13, 14). The changes in drinks per week observed may not have been large enough to exceed a threshold above which the alcohol risk level changes. However, data of the development of alcohol risk level in the PRINT control group showed that 23% of the low-risk drinkers had increased to at-risk level during one year (41). Another explanation may be that the category of low-risk drinking obscures important patterns of low alcohol use, and that persons with different patterns develop differently over time. Relatedly, there may be a mixing of low-risk drinkers with infrequent heavy episodic drinkers (12). In addition to that, reactivity to alcohol assessment measures may partly explain the increase in alcohol use (42, 43). That is, participants may start to pay closer attention to their alcohol use after the first assessment, and may either remember it more accurately at later assessments (i.e., lower level of underreporting) or actually change it.

The current study also revealed that there may be different response patterns to individualized alcohol feedback among persons in different trajectory classes. For example, baseline at-risk drinkers with a *light-stable* trajectory benefitted in the long-term. This is in line with previous studies which have found that the effects of motivation-enhancing brief alcohol interventions in proactively recruited samples increase over time (44, 45). Low drinking amounts are likely to be associated with low motivation to change (39), and the intervention may require time to produce changes on the behavior level in these groups. For low-risk drinkers in the *very light-slightly increasing* class, a beneficial intervention effect was observed at month 6, although the difference appears small and clinical relevance may be questionable. The finding is in line with 12-month outcome data of the PRINT trial published elsewhere (19) showing medium-term benefits of the intervention among low-risk drinkers but not among at-risk drinkers. For baseline at-risk drinkers in the *medium-stable* class, assessment only seems to work better than individualized motivation-enhancing feedback. Although findings were not statistically significant, the feedback regarding their alcohol use, motivation to change, pros and cons of alcohol use, self-efficacy, and processes of change may have produced resistance to change in in the *medium-stable* class. The same may apply in the short-term to persons in the *high-decreasing* class who initially increased their alcohol use to a higher extent when they received feedback compared to no intervention. The adverse effect was then reversed to a beneficial one and attenuated after the intervention activities have ended. Much remains to be learned about the motivational processes behind the initiation and maintenance of behavior change, especially but not exclusively in population groups with low drinking amounts.

With respect to the association of alcohol trajectories with socio-demographic variables, our results support previous findings of trajectories of high or increasing alcohol use among males, current smokers, and persons with a high level of school education (14, 46–48). Among at-risk drinkers, older age increased the probability to belong to the trajectory class with highest average amounts of alcohol use. This is in line with findings from population-based cohort studies showing that daily or near daily alcohol use became common during mid to older age, especially among men (49, 50).

This study capitalized on a proactively recruited general population sample of adults with alcohol use that has been followed-up over 3 years. However, it should be noted that the current analysis is exploratory, and our findings should be interpreted with caution. Five limitations need to be considered. First, although more than two third of the target population were reached, selection bias may have limited the generalizability for the whole population of adults with alcohol use. The current sample had a high representation of persons with high socioeconomic position as indicated by years of school education and employment status. This can partly be explained by the socio-demographic structure of the Greifswald population characterized by a large proportion of university employees and students. Second, trajectories in the control group do not necessarily represent natural history and may also be explained by reactivity, regression to the mean, or other methodological issues. Such effects are hard to quantify and disentangle properly from “true” trajectories. Conventional attempts to do so, such as testing linear trends over time and intercept-slope correlations as indicators for reactivity and regression effects, respectively, are likely to overestimate the extent of change explained by methodological artifacts. The opportunities offered by latent variable models promise some progress in this field. This approach allows one, for example, to test for measurement invariance within a confirmatory factor analysis framework or to account for method effects by latent residual factors. Third, our findings should not be interpreted as unequivocal evidence for or against intervention effects in subgroups due to power and multiple comparison problems. The idea behind the current study was rather to explore beneficial or adverse effects of the intervention, when these effects are likely to appear and for whom. Fourth, although mixture modeling was used to identify subgroups of persons with homogenous trajectories of alcohol use, it still focused on mean trajectories within classes, and there may be variation around the mean trajectories. Fifth, entropy values indicate that some classes are not perfectly distinguishable by the data (e.g., *light-stable* vs. *medium-stable* or *very light-slightly increasing* vs. *light-increasing*), although average class posterior probabilities indicate acceptable clarity in classification.

Although preliminary, our findings have three important implications for future studies in the field of alcohol prevention. First, persons invited to participate in interventions that intend to have population impact should be unselected in relation to their alcohol use. A risk threshold for alcohol is no longer in accordance with the current literature (8–11). Our study demonstrated that adult low-risk drinkers are reachable for intervention efforts to reduce alcohol use, they were adherent to the intervention, and there is cautious evidence of long-term benefit among subgroups of persons with low drinking amounts. Second, interventions should be selected on the basis of longitudinal trajectories of alcohol use, not on the basis of a single screening for alcohol, not least for reasons of temporal variations of alcohol use and potential misclassification (12, 41). It is conceivable that participants are allocated to different trajectory classes based on repeated alcohol assessments before the intervention starts, as suggested by Muthén et al. (16). Alternatively, they could receive an intervention based on the first assessment and adjustments can be made if appropriate when more data is available (e.g., change in alcohol use or motivation, intervention benefit). Third, the effectiveness of interventions should be tested on the basis of trajectories of alcohol use, not on the basis of a static measure at a single moment in time. Ideally, the outcome refers to trajectories over the life course. This requires much longer follow-up periods than is currently the case in brief intervention trials.

This study provides first insights into the existence of a priori unknown trajectories of at-risk and low-risk alcohol use over 3 years with and in absence of a low-cost brief intervention for alcohol. Our data indicate long-term intervention effects in some subgroups, with greater benefits among groups of persons with low amounts of alcohol. Although research is needed to replicate our findings, they may help to identify refinements in the development of individualized interventions to reduce alcohol use in the population.

## Data availability statement

The raw data supporting the conclusions of this article will be made available by the authors, without undue reservation.

## Ethics statement

The studies involving human participants were reviewed and approved by Ethics Committees of University Medicine Greifswald and TU Dresden. The patients/participants provided their written informed consent to participate in this study.

## Author contributions

SB: conceptualization, data curation, formal analysis, funding acquisition, investigation, and methodology, project administration, software, supervision, validation, and visualization. AS: data curation, formal analysis, investigation, methodology, project administration, software, supervision, and validation. JF-A: conceptualization, methodology, and software. MZ: data curation and investigation. GB: software. CM: investigation. UJ: conceptualization and methodology. All authors contributed to the article and approved the submitted version.

## Funding

The study was funded by the German Research Foundation (BA 5858/2-1; BA 5858/2-3).

## Conflict of interest

The authors declare that the research was conducted in the absence of any commercial or financial relationships that could be construed as a potential conflict of interest.

## Publisher's note

All claims expressed in this article are solely those of the authors and do not necessarily represent those of their affiliated organizations, or those of the publisher, the editors and the reviewers. Any product that may be evaluated in this article, or claim that may be made by its manufacturer, is not guaranteed or endorsed by the publisher.
