# Supplementary material for: Three-year trajectories of alcohol use among at-risk and among low-risk drinkers in a general population sample of adults: A latent class growth analysis of a brief intervention trial
Source: Front Public Health. 2022 Nov 17;10:1027837. doi: 10.3389/fpubh.2022.1027837 (PMC9714030; doi:10.3389/fpubh.2022.1027837)
Supplement: Supplementary file 1 [file Table_1.DOCX]

**Table S1** Study group differences (net changes) in drinks per week over 3 years according to trajectory class (model without covariates).

|  | At-risk drinkers | | | | | | Low-risk drinkers | | | |
| --- | --- | --- | --- | --- | --- | --- | --- | --- | --- | --- |
|  | Light-stable | | Medium-stable | | High-decreasing | | Very light-slightly increasing | | Light-increasing | |
|  | IRR | (95% CI) | IRR | (95% CI) | IRR | (95% CI) | IRR | (95% CI) | IRR | (95% CI) |
| Month 3 | 0.94 | (0.75–1.18) | 0.92 | (0.70–1.20) | 0.80 | (0.49–1.29) | 1.02 | (0.74–1.43) | 1.04 | (0.77–1.41) |
| Month 6 | 1.02 | (0.72–1.44) | 0.79 | (0.59–1.07) | 1.37 | (0.71–2.62) | **1.74** | **(1.19–2.55)** | 1.09 | (0.83–1.44) |
| Year 1 | 1.06 | (0.73–1.53) | 0.83 | (0.62–1.11) | 1.14 | (0.44–2.93) | 1.09 | (0.74–1.60) | 0.91 | (0.69–1.19) |
| Year 3 | **1.93** | **(1.15–3.23)** | 0.72 | (0.45–1.13) | 0.99 | (0.51–1.91) | 0.89 | (0.62–1.28) | 1.16 | (0.81–1.67) |
| IRR, incidence rate ratio; CI, confidence interval. | | | | | | | | | | |
