# Supplementary figures and images for: Three-year trajectories of alcohol use among at-risk and among low-risk drinkers in a general population sample of adults: A latent class growth analysis of a brief intervention trial
Source: Front Public Health. 2022 Nov 17;10:1027837. doi: 10.3389/fpubh.2022.1027837 (PMC9714030; doi:10.3389/fpubh.2022.1027837)

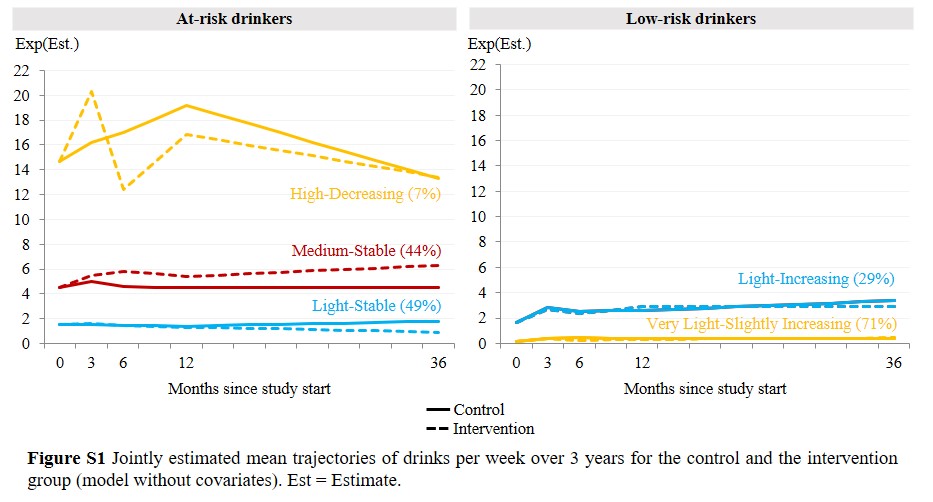

Supplement: Supplementary file 2 [file Image_1.jpg]
